# Patterns and timing of sunlight exposure and risk of basal cell and squamous cell carcinomas of the skin – a case–control study

**DOI:** 10.1186/1471-2407-12-417

**Published:** 2012-09-20

**Authors:** Michelle R Iannacone, Wei Wang, Heather G Stockwell, Kathleen O’Rourke, Anna R Giuliano, Vernon K Sondak, Jane L Messina, Richard G Roetzheim, Basil S Cherpelis, Neil A Fenske, Dana E Rollison

**Affiliations:** 1 Department of Cancer Epidemiology, Moffitt Cancer Center, Tampa, FL, USA; 2 Department of Epidemiology and Biostatistics, College of Public Health, University of South Florida, Tampa, FL, USA; 3 Cutaneous Oncology Program, Moffitt Cancer Center, Tampa, FL, USA; 4 Department of Pathology and Cell Biology, University of South Florida College of Medicine, Tampa, FL, USA; 5 Department of Dermatology, University of South Florida College of Medicine, Tampa, FL, USA; 6 Department of Cutaneous Surgery, University of South Florida College of Medicine, Tampa, FL, USA; 7 Department of Family Medicine, University of South Florida College of Medicine, Tampa, FL, USA; 8 Infections and Cancer Biology Group, International Agency for Research on Cancer, 150 cours Albert-Thomas, 69372, Lyon Cedex 08, France

**Keywords:** Case–control, Sunlight, Patterns, Basal cell carcinoma, Squamous cell carcinoma, Non-melanoma skin cancer

## Abstract

**Background:**

Non-melanoma skin cancer (NMSC), comprised of basal (BCC) and squamous (SCC) cell carcinomas, is the most common cancer in Caucasians. Ultraviolet radiation (UVR) exposure is the most important environmental risk factor for NMSC. However, the precise relationship between UVR and the risk of NMSC is complex, and the relationship may differ by skin cancer type.

**Methods:**

A case–control study was conducted among Florida residents to investigate measures of patterns (intermittent vs. continuous) and timing (childhood vs. adulthood) of sunlight exposure in BCC and SCC. Participants included 218 BCC and 169 SCC cases recruited from a university dermatology clinic and 316 controls with no history of skin or other cancers.

**Results:**

A history of blistering sunburn (a measure of intermittent sunlight exposure) was associated with both BCC (OR = 1.96, 95% CI = 1.27-3.03) and SCC (OR = 2.02, 95% CI = 1.22-3.33). Additionally, having a job in the sun for ≥3 months for 10 years or longer (a measure of continuous sunlight exposure) was also associated with both BCC and SCC in our study population. With the exception of younger age at first blistering sunburn, measures of younger age at sunlight exposure tended to be associated with SCC, but not BCC risk.

**Conclusions:**

Results from the current study suggest that sunlight exposure is associated with both BCC and SCC risk regardless of the pattern in which the exposure was received (i.e. intermittent vs. continuous). The data also suggest that sunlight exposure at a younger age may be more important for SCC but not BCC, however additional studies are needed to further characterize sunlight exposure-response relationships in different types of NMSC.

## Background

Non-melanoma skin cancer (NMSC), comprised of basal cell (BCC) and squamous cell (SCC) carcinomas, is the most common cancer in Caucasians, with more than one million new cases diagnosed annually in the United States (U.S.) alone [1]. While the mortality associated with NMSC is low [2,3], patients with multiple NMSC’s may experience substantial morbidity, and treatment costs for NMSC are high at the national level [4]. Furthermore, a history of NMSC has been consistently associated with increased risk of subsequent primary cancers of other sites in studies from both the U.S. and Europe [5-13].

Ultraviolet radiation (UVR) exposure is considered the most important environmental risk factor for both BCC and SCC. However, the precise relationship between UVR and the risk of NMSC is complex, and the relationship may differ by skin cancer type. Starting in the late 1950s, researchers began to identify total (cumulative) outdoor sunlight exposure hours and sunlight exposure on working and non-working days [14-19] as risk factors for NMSC. Results from these studies suggested that BCC and SCC may have different exposure-response relationships with sunlight.

Patterns of sunlight exposure are continuous (i.e. persons working outdoors or living in a geographic region with a high annual UV index) or intermittent (i.e. persons working indoors and experiencing most of their sunlight exposure on the weekends or while vacationing to regions with a higher UV index than their place of residence). Timing of sunlight exposure refers to the period of life during which the majority of a person’s sunlight exposure was experienced: childhood/adolescence, adulthood or both. Evidence from previous studies suggests that intermittent and childhood sunlight exposure may be important for the pathogenesis of BCC, whereas continuous, lifelong sunlight exposure may be important for SCC [20-24].

To further understand the relationship between sunlight exposure and risk of NMSC, a case–control study was conducted to investigate measures of patterns (intermittent vs. continuous) and timing (childhood vs. adulthood) of sunlight exposure in BCC and SCC. A major limitation of previously published studies is that they do not present direct comparisons between BCC and SCC from the same study population for associations with measures of patterns and timing of sunlight exposure. Therefore, differences in the observed associations may be explained by methodological inconsistencies in exposure measurement between study populations that investigate BCC or SCC alone. This is the first case–control study to simultaneously evaluate identical measures of patterns and timing of sunlight exposure as they are related to both BCC and SCC in the same U.S. population with high annual UVR exposure. The goal of the current study was to identify potential differences or similarities in sunlight exposure responses for BCC and SCC risk.

## Methods

### Study design and population

A clinic-based case–control study was conducted to evaluate the relationship between patterns and timing of sunlight exposure and risk of BCC and SCC. Complete study procedures have been described in detail elsewhere [25]. The University of South Florida (USF) Dermatology (D) clinic served as the primary location for recruitment of NMSC cases, comprised of patients with histologically-confirmed BCC or SCC. Control participants were recruited from the USF Family Medicine (FM) clinic and Moffitt’s Lifetime Cancer Screening & Prevention (LCS) clinic. Controls were individuals who self-reported no history of skin or other types of cancer and underwent a skin cancer screening exam at the time of study enrollment and screened negative for skin cancer. Additionally, patients that screened positive for a suspicious lesion, underwent a biopsy and were determined to be negative for skin cancer were also included as controls. All participants were recruited between October 30, 2006 and December 24, 2008. All participants provided written informed consent, and all study procedures were approved by the institutional review board at the University of South Florida.

Participation rates for the USF-D, the USF-FM, and LCS clinics were 80%, 47%, and 65%, respectively. There were no statistically significant differences in age or gender between those NMSC patients who agreed to participate and those that refused. A total of 358 controls, 245 BCC cases and 191 SCC cases were enrolled. The current study population was restricted to participants that provided complete questionnaire data (Controls: n = 358; BCC: n = 222; SCC: n = 170) and to White individuals and includes 218 BCC and 169 SCC cases and 316 controls, between the ages of 18 and 80.

### Exposure assessment

Self-administered questionnaires were used to obtain information on sunlight exposures and potential confounding factors, including age, gender, ethnicity, education, eye and hair color, ever smoking, skin sensitivity to sunlight exposure (measured by skin reaction to one hour of sunlight exposure for the first time without sunscreen), and tanning ability (measured by change in skin color to repeated exposure to the summer sun). Patterns of sunlight exposure were measured using questions on history of blistering sunburn (yes/no), ever having a job in the sunlight for ≥3 months (yes/no), the number of years with a job in the sunlight for ≥3 months (<1, 1–5, 6–10, or >10 years), lifetime frequency of tanning bed use (≤10, 11–50, 51–100, >100 times), frequency of sunscreen application with a sunlight protection factor (SPF) of ≥15 when outside for more than 15 minutes during the summer (always, often, sometimes, rarely, never), and the number of hours of mid-day sunlight exposure on a typical weekday (<1, 1–2, 3–4, 5–6 hours) and weekend day (<1, 1–2, 3–4, 5–6 hours) in the summer during one’s teen years, twenties, thirties, and the past ten years prior to study enrollment. Experiencing blistering sunburn is considered a marker of intermittent sunlight exposure. Additionally, using sunscreen always/often or rarely/never is considered experiencing continuous sunlight exposure and using sunscreen some of time is considered intermittent sunlight exposure.

Timing of sunlight exposure was measured using questions on the age at which a blistering sunburn was experienced (≤5, 6–10, 11–15, 16–20, >20 years), the number of moles larger than one quarter of an inch in diameter on the forearms (none, <10, 10–25, >25 moles) and on the entire body (none, <10, 10–25, >25 moles), the age at first tanning bed use (≤15, 16–20, >20 years), and the number of hours of mid-day sunlight exposure on a typical weekday (<1, 1–2, 3–4, 5–6 hours) and weekend day (<1, 1–2, 3–4, 5–6 hours) in the summer during one’s teen years, twenties, thirties, and in the past ten years prior to study enrollment. The presence of moles is considered an indicator of increased sunlight exposure in childhood or adolescence [26-31].

### Statistical analysis

Demographic and skin cancer risk factors were compared between cases and controls using the chi-square test. To test whether measures of patterns or timing of sunlight exposure were associated with BCC or SCC, separate odds ratios (OR) and corresponding 95% confidence intervals (CI) for each skin cancer type were calculated using unconditional logistic regression. Backward stepwise elimination was used to identify confounders from those factors previously shown to be associated with sunlight exposure and NMSC, including age (as a continuous variable), gender, ethnicity, education, eye, hair, and un-tanned skin color, cutaneous sensitivity and tanning ability to sunlight exposure, history of ever smoking, and alcohol consumption in the past year. Each factor retained in the model at p < .10 was included in the final regression models; these factors included age, gender, ethnicity, education, eye and hair color, cutaneous sensitivity, tanning ability, and history of ever smoking. Variance inflation factors and Pearson correlation coefficients were estimated to identify multicollinear relationships between independent risk factors. No collinearity between co-factors and measures of patterns and timing of sunlight exposure was observed. Tests for trend in associations between measures of patterns and timing of sunlight exposure in BCC/SCC were conducted by assigning ordinal values to each category and including the ordinal variable in the logistic regression model.

Factors associated with skin susceptibility to sunlight exposure have the potential to be factors on the causal pathway between UVR exposure and skin cancer. Therefore, to demonstrate the impact of these factors on the associations of interest, we present results from two different multivariate analyses. The first multivariate analysis adjusted for demographic and lifestyle factors only (i.e. age, gender, education, and history of ever smoking) and the second adjusted for demographic and lifestyle factors, as well as measures of skin susceptibility to sunlight exposure (i.e. ethnicity, eye and hair color, cutaneous sensitivity and tanning ability to sunlight exposure).

Utilizing data collected on the number of hours of sunlight exposure experienced on a typical weekday and weekend day during the summer in different time periods, summary scores were calculated. To measure cumulative sunlight exposure in *early life* (i.e. teens, twenties, and thirties), a median value was applied to each category of hours of sunlight exposure (<1 hour = 0.5; 1–2 hours = 1.5; 3–4 hours = 3.5; 5–6 hours = 5.5) on a weekday and weekend day. The median values for weekday and weekend sunlight exposure were first summed for each age group, then summed across age groups (i.e. teens, twenties, and thirties) and finally divided into three categories: low, medium, and high. For intermittent sunlight exposure in *early life*, median values were once again applied to each category of hours of sunlight exposure. The ratio of median hours on a weekend day relative to that on a weekday was estimated separately for one’s teen years, twenties, and thirties, summed across the three decades, and then divided into three groups: low (representing continuous sunlight exposure), medium, and high. Analyses including summary scores measuring sunlight exposure in *early life* were restricted to participants who were ≥40 years of age.

For patterns of sunlight exposure by age at exposure (i.e. one’s teens, twenties, thirties, and the 10 years prior to study enrollment), the participant was considered as having had *continuous sunlight exposure* if the reported number of hours of weekday sunlight exposure (1–2 or 3–6 hours) equaled that of weekend sunlight exposure (1–2 or 3–6 hours). However, if the reported number of hours of weekday sunlight exposure was less than that of weekend sunlight exposure, then the participant was considered as having *intermittent* sunlight exposure. Participants classified as having *continuous* or *intermittent* sunlight exposure were compared to participants with <1 hour of sunlight exposure on a typical weekday and weekend day. Daily sunlight exposure by age at exposure was measured by summing the median values of weekday and weekend hours of sunlight exposure and then dividing the values into three categories: low, medium, and high, independently for each time period.

The likelihood ratio test (LRT) was used to compare the statistical significance of the differences in effect sizes between BCC and SCC for each sun-related factor measured. Multiple testing was accounted for using Bonferroni correction. All analyses were performed using the SAS statistical software package (version 9.1.3; SAS Institute).

## Results

Demographic, lifestyle, and skin susceptibility factors are presented for cases and controls in Table 1. Compared to controls, cases were significantly more likely to be older in age (BCC: p = <.0001; SCC: p = <.0001), male (BCC: p = <.0001; SCC: p = <.0001), less educated (BCC: p = 0.0004; SCC: p = 0.001), and ever smokers (BCC: p = 0.002; SCC: p = <.0001). Additionally, NMSC cases were more likely to have light eye and hair color, a greater tendency to burn and a lesser tendency to tan from sunlight exposure, compared to controls.

**Table 1:** Demographic, lifestyle, and skin cancer risk factors in BCC and SCC cases and controls

	**Controls (n = 316)**	**BCC (n=218)**		**SCC (n = 169)**	
**Variable**	**n (%)**	**n (%)**	**p-value**^**1**^	**n (%)**	**p-value**^**1**^
Age mean (S.D.)	55.6 (11.8)	62.8 (11.9)	<.0001	64.8 (9.6)	<.0001
Age (years)					
18–29	9 (2.9)	1 (0.5)	<.0001	1 (0.6)	<.0001
30–39	21 (6.7)	6 (2.8)		2 (1.2)	
40–49	55 (17.4)	24 (11.0)		10 (5.9)	
50–59	109 (34.5)	46 (21.1)		30 (17.8)	
60–69	88 (27.9)	64 (29.4)		68 (40.2)	
70–80	34 (10.8)	77 (35.3)		58 (34.3)	
Gender					
Male	117 (37.0)	133 (61.0)	<.0001	108 (63.9)	<.0001
Female	199 (63.0)	85 (39.0)		61 (36.1)	
Ethnicity					
Non-Hispanic	280 (88.6)	208 (95.4)	0.003	161 (95.3)	0.0003
Hispanic	32 (10.1)	7 (3.2)		2 (1.2)	
Education					
≤12 years	32 (10.1)	46 (21.1)	0.0004	36 (21.3)	0.001
>12 years	280 (88.6)	168 (77.1)		129 (76.3)	
Smoked 100 cigarettes					
Never	161 (50.9)	81 (37.2)	0.002	51 (30.2)	<.0001
Ever	154 (48.7)	134 (61.5)		114 (67.5)	
Alcohol consumption					
≥ 1 drink in past year	274 (86.7)	177 (81.2)	0.09	130 (76.9)	0.02
No drinks in past year	40 (12.7)	39 (17.9)		35 (20.7)	
Eye color					
Blue	94 (29.7)	87 (40.0)	0.009	69 (40.8)	0.02
Green	50 (16.1)	24 (11.0)		25 (14.8)	
Hazel	52 (16.5)	48 (22.0)		31 (18.3)	
Light brown	36 (11.4)	22 (10.1)		18 (10.7)	
Dark brown	80 (25.3)	35 (16.1)		22 (13.0)	
Hair Color					
Black/Brown	245 (77.5)	152 (69.7)	0.04	113 (66.9)	0.02
Blonde/Red	70 (22.2)	65 (29.8)		53 (31.4)	
Color of un-tanned skin					
White	299 (94.9)	209 (96.3)	0.38	161 (95.3)	0.55
Brown	15 (4.8)	7 (3.2)		6 (3.6)	
Cutaneous sensitivity to sunlight exposure					
Sunburn with blisters	29 (9.2)	33 (15.1)	0.0001	22 (13.0)	0.005
Sunburn w/o blisters	96 (30.4)	95 (43.6)		71 (42.0)	
Mild sunburn/tan	144 (45.6)	65 (29.8)		50 (29.6)	
Tan/no change color	44 (13.9)	21 (9.6)		22 (13.0)	
Tanning ability to sunlight exposure					
It is unable to tan	22 (7.0)	15 (6.9)	0.04	26 (15.4)	<.0001
Tan if you work at it	103 (32.6)	93 (42.7)		77 (45.6)	
It tans easily	186 (58.9)	104 (47.7)		62 (37.6)	

^1^p-value for chi-square test.

Associations between patterns of sunlight exposure and NMSC are presented in Table 2. When adjusting for demographic and lifestyle factors only, a history of blistering sunburn was positively associated with both BCC (OR = 1.96, 95% CI = 1.27-3.03) and SCC (OR = 2.02, 95% CI = 1.22-3.33). Ever having a job in the sunlight for ≥3 months was significantly associated with SCC (OR = 1.73, 95% CI = 1.06-2.83) but not BCC (OR = 1.38, 95% CI = 0.89-2.14). However, having a job in the sunlight for ≥3 months for >10 years was significantly associated with both BCC (OR = 2.14, 95% CI = 1.12-4.11) and SCC (OR = 2.54, 95% CI = 1.23-5.28). With the exception of having a job in the sunlight for >10 years, the associations described above were no longer statistically significant after skin susceptibility co-factors were added to the multivariate models. When adjusting for demographic and lifestyle factors only, no associations were observed between levels of cumulative sunlight exposure or patterns of exposure in one’s twenties or thirties and either BCC or SCC. However, after additional adjustment for measures of skin susceptibility, high levels of cumulative sunlight exposure were associated with BCC (OR = 1.88, 95% CI = 1.07-3.31) and medium (OR = 2.36, 95% CI = 1.22-4.57) and high (OR = 2.47, 95% CI = 1.25-4.91) levels of cumulative sunlight exposure were significantly associated with SCC, compared to low levels in early life. Additionally, sunlight exposure in one’s twenties was associated with SCC regardless of the pattern of exposure; specifically, an OR of 2.99 (95% CI = 1.19-7.48) was associated with continuous hours and an OR of 3.15 (95% CI = 1.27-7.83) was associated with intermittent hours of exposure compared to <1 hour of sunlight exposure. Finally, in one’s thirties, statistically significant associations were observed between intermittent hours of sunlight exposure and BCC (OR = 2.09, 95% CI = 1.11-3.93) while continuous hours of sunlight exposure were associated with SCC (OR = 2.25, 95% CI = 1.02-4.94), compared to <1 hour of exposure, when adjusting for skin susceptibility co-factors. Regardless of the covariates included in the multivariate models, no statistically significant associations in BCC or SCC were observed with tanning bed use, sunscreen use, levels of intermittent sunlight exposure in early life, and patterns of sunlight exposure in one’s teens and the past ten years prior to study enrollment.

**Table 2:** Associations of measures of patterns of sunlight exposure in BCC and SCC cases and controls

	**Controls (n = 316)**	**Basal cell carcinoma (n = 218)**			**Squamous cell carcinoma (n = 169)**		
**Variable**	**n (%)**	**n (%)**	**OR (95% CI)**^**1**^	**OR (95% CI)**^**2**^	**n (%)**	**OR (95% CI)**^**1**^	**OR (95% CI)**^**2**^
Blistering Sunburn							
No	101 (32.3)	54 (25.0)	1.00 (reference)	1.00 (reference)	38 (23.0)	1.00 (reference)	1.00 (reference)
Yes	212 (67.7)	162 (75.0)	1.96 (1.27-3.03)	1.56 (0.96-2.54)	127 (77.0)	2.02 (1.22-3.33)	1.24 (0.71-2.18)
Job in sun ≥3 months							
No	227 (72.8)	120 (55.3)	1.00 (reference)	1.00 (reference)	86 (51.8)	1.00 (reference)	1.00 (reference)
Yes	85 (27.2)	97 (44.7)	1.38 (0.89-2.14)	1.31 (0.81-2.12)	80 (48.2)	1.73 (1.06-2.83)	1.72 (0.99-2.97)
# years with job							
≤10	57 (18.7)	47 (22.3)	1.17 (0.70-1.94)	1.07 (0.61-1.86)	44 (27.0)	1.64 (0.94-2.86)	1.64 (0.88-3.07)
>10	21 (6.9)	44 (20.9)	2.14 (1.12-4.11)	2.12 (1.05-4.27)	33 (20.2)	2.54 (1.23-5.28)	2.36 (1.07-5.20)
			p_trend_ = 0.03	p_trend_ = 0.06		p_trend_ = 0.01	p_trend_ = 0.02
Lifetime tanning bed use	209 (71.3)	175 (82.5)	1.00 (reference)	1.00 (reference)	127 (80.9)	1.00 (reference)	1.00 (reference)
Never used	44 (15.0)	25 (11.8)	0.99 (0.56-1.76)	0.99 (0.53-1.82)	18 (11.5)	1.01 (0.52-1.98)	0.80 (0.38-1.71)
1–10 times	40 (13.7)	12 (5.7)	0.64 (0.30-1.36)	0.64 (0.30-1.36)	12 (7.6)	1.67 (0.75-3.73)	1.85 (0.74-4.62)
>10 times			p_trend_ = 0.32	p_trend_ = 0.20		p_trend_ = 0.29	p_trend_ = 0.40
Apply SPF^3^ ≥15							
Always/often	124 (39.6)	82 (37.8)	1.00 (reference)	1.00 (reference)	53 (32.5)	1.00 (reference)	1.00 (reference)
Sometimes	98 (31.3)	59 (27.2)	0.79 (0.50-1.26)	0.87 (0.52-1.45)	56 (34.4)	0.83 (0.49-1.42)	0.86 (0.47-1.59)
Rarely/never	91 (29.1)	76 (35.0)	0.83 (0.52-1.32)	0.93 (0.56-1.54)	54 (33.1)	0.79 (0.46-1.36)	0.87 (0.48-1.60)
Cumulative sunlight exposure							
Low	87 (32.3)	52 (27.4)	1.00 (reference)	1.00 (reference)	35 (22.7)	1.00 (reference)	1.00 (reference)
Medium	92 (34.2)	54 (28.4)	1.02 (0.61-1.69)	1.42 (0.81-2.50)	57 (37.0)	1.49 (0.84-2.64)	2.36 (1.22-4.57)
High	90 (33.5)	84 (44.2)	1.37 (0.83-2.27)	1.88 (1.07-3.31)	62 (40.3)	1.59 (0.88-2.87)	2.47 (1.25-4.91)
Intermittent sunlight exposure							
Low	91 (33.8)	80 (42.1)	1.00 (reference)	1.00 (reference)	59 (38.3)	1.00 (reference)	1.00 (reference)
Medium	84 (31.2)	52 (27.4)	0.99 (0.60-1.64)	1.26 (0.72-2.22)	46 (29.9)	1.25 (0.71-2.20)	1.58 (0.83-3.00)
High	94 (34.9)	58 (30.5)	1.15 (0.70-1.88)	1.23 (0.72-2.10)	49 (31.8)	1.48 (0.85-2.58)	1.57 (0.83-2.94)
Patterns by age at exposure							
Teens							
<1 hour	18 (6.0)	12 (6.0)	1.00 (reference)	1.00 (reference)	4 (2.5)	1.00 (reference)	1.00 (reference)
Continuous hours	151 (50.0)	113 (56.8)	1.04 (0.45-2.41)	0.97 (0.38-2.48)	96 (60.4)	2.33 (0.69-7.90)	1.76 (0.48-6.47)
Intermittent hours	133 (44.0)	74 (37.2)	1.08 (0.46-2.54)	1.10 (0.42-2.83)	59 (37.1)	2.26 (0.66-7.78)	1.86 (0.50-6.94)
Twenties							
<1 hour	34 (11.3)	18 (9.0)	1.00 (reference)	1.00 (reference)	11 (6.9)	1.00 (reference)	1.00 (reference)
Continuous hours	102 (33.9)	91 (45.5)	1.36 (0.68-2.71)	1.58 (0.75-3.36)	74 (46.5)	2.01 (0.86-4.67)	2.99 (1.19-7.48)
Intermittent hours	165 (54.8)	91 (45.5)	1.30 (0.66-2.56)	1.56 (0.74-3.26)	74 (46.5)	2.11 (0.92-4.88)	3.15 (1.27-7.83)
Thirties							
<1 hour	60 (20.5)	27 (13.6)	1.00 (reference)	1.00 (reference)	18 (11.3)	1.00 (reference)	1.00 (reference)
Continuous hours	85 (29.0)	79 (39.9)	1.31 (0.72-2.40)	1.77 (0.90-3.49)	69 (43.4)	1.55 (0.77-3.10)	2.25 (1.02-4.94)
Intermittent hours	148 (50.5)	92 (46.5)	1.38 (0.79-2.41)	2.09 (1.11-3.93)	72 (45.3)	1.47 (0.76-2.85)	1.95 (0.92-4.12)
Past 10 years							
<1 hour	63 (28.6)	52 (30.2)	1.00 (reference)	1.00 (reference)	49 (33.1)	1.00 (reference)	1.00 (reference)
Continuous hours	74 (33.6)	83 (48.3)	0.88 (0.51-1.52)	1.14 (0.62-2.12)	64 (43.2)	0.81 (0.46-1.42)	1.35 (0.69-2.64)
Intermittent hours	83 (37.7)	37 (21.5)	0.57 (0.32-1.03)	0.67 (0.35-1.28)	35 (23.6)	0.60 (0.33-1.10)	0.93 (0.46-1.89)

^1^Odds ratios (OR) and 95% confidence intervals (CI) adjusted for age, gender, education, and history of ever smoking.

^2^OR and 95% CI adjusted for age, gender, education, history of ever smoking, ethnicity, eye and hair color, cutaneous sensitivity and tanning ability to sunlight exposure.

^3^SPF = sun protection factor.

Table 3 presents the associations between measures of timing of sunlight exposure and BCC and SCC. When adjusting for demographic and lifestyle factors only, associations with SCC were observed for the presence of ≥10 moles on the forearms (OR = 3.27, 95% CI = 1.12-9.58) and entire body (OR = 2.12, 95% CI = 1.11-4.06), compared to no moles. Similar associations were not observed with BCC. Experiencing a blistering sunburn in young childhood or adolescence was significantly associated with both BCC (<10 years: OR = 1.97, 95% CI = 1.14-3.42; 10–20 years: OR = 2.15, 95% CI = 1.32-3.52) and SCC (<10 years: OR = 2.25, 95% CI = 1.22-4.13; 10–20 years: OR = 2.37, 95% CI = 1.34-4.21), compared to never experiencing blistering sunburn. SCC cases were more likely to begin using a tanning bed prior to age 20 (OR = 1.97, 95% CI = 1.01-3.85), compared to never users. No significant associations with BCC were observed for age at first tanning bed use. Elevated OR estimates were observed for high levels of daily sunlight exposure during the summer with BCC and SCC across all time periods, however, none of these associations achieved statistical significance (Table 3). When including measures of skin susceptibility to sunlight exposure to the multivariate models, little differences were observed in the magnitudes of associations between measures of timing of sunlight exposure and BCC/SCC.

**Table 3:** Associations of measures of timing of sunlight exposure in BCC and SCC cases and controls

	**Controls (n = 316)**	**Basal cell carcinoma (n = 218)**			**Squamous cell carcinoma (n = 169)**		
**Variable**	**n (%)**	**n (%)**	**OR (95% CI)**^**1**^	**OR (95% CI)**^**2**^	**n (%)**	**OR (95% CI)**^**1**^	**OR (95% CI)**^**2**^
# of moles on forearms							
None	220 (71.4)	155 (71.8)	1.00 (reference)	1.00 (reference)	115 (69.7)	1.00 (reference)	1.00 (reference)
<10	80 (26.0)	53 (24.5)	0.84 (0.54-1.31)	0.65 (0.40-1.06)	39 (23.6)	0.92 (0.56-1.52)	0.94 (0.54-1.64)
≥10	8 (2.6)	8 (3.7)	1.65 (0.57-4.77)	1.75 (0.55-5.61)	11 (6.7)	3.27 (1.12-9.58)	2.69 (0.75-9.59)
			p_trend_ = 0.96	p_trend_ = 0.45		p_trend_ = 0.25	p_trend_ = 0.43
# of moles on entire body							
None	118 (39.1)	79 (36.9)	1.00 (reference)	1.00 (reference)	56 (34.1)	1.00 (reference)	1.00 (reference)
<10	147 (48.7)	107 (50.0)	1.11 (0.73-1.67)	1.03 (0.66-1.60)	76 (46.3)	1.15 (0.72-1.86)	1.22 (0.71-2.09)
≥10	37 (12.3)	28 (13.1)	1.18 (0.64-2.19)	1.06 (0.55-2.04)	32 (19.5)	2.12 (1.11-4.06)	2.16 (1.03-4.52)
			p_trend_ = 0.54	p_trend_ = 0.86		p_trend_ = 0.04	p_trend_ = 0.06
Age at 1^st^ blistering sunburn							
None	101 (32.9)	54 (25.2)	1.00 (reference)	1.00 (reference)	38 (23.5)	1.00 (reference)	1.00 (reference)
<10 years	62 (20.2)	47 (22.0)	1.97 (1.14-3.42)	1.35 (0.73-2.49)	46 (28.4)	2.25 (1.22-4.13)	1.07 (0.53-2.15)
10–20 years	108 (35.2)	84 (39.3)	2.15 (1.32-3.52)	1.73 (1.00-2.99)	65 (40.1)	2.37 (1.34-4.21)	1.62 (0.86-3.04)
>20 years	36 (11.7)	29 (13.6)	1.71 (0.89-3.28)	1.67 (0.83-3.37)	13 (8.0)	0.96 (0.42-2.20)	0.81 (0.33-2.01)
			p_trend_ = 0.01	p_trend_ = 0.05		p_trend_ = 0.17	p_trend_ = 0.53
Age at 1^st^ tanning bed use							
Never used	209 (67.0)	175 (80.3)	1.00 (reference)	1.00 (reference)	127 (76.5)	1.00 (reference)	1.00 (reference)
≤20 years	38 (12.2)	20 (9.2)	1.09 (0.58-2.06)	1.10 (0.55-2.18)	23 (13.9)	1.97 (1.01-3.85)	1.97 (0.91-4.27)
>20 years	65 (20.8)	23 (10.6)	0.64 (0.64-1.12)	0.56 (0.30-1.05)	16 (9.6)	0.77 (0.40-1.50)	0.78 (0.37-1.65)
			p_trend_ = 0.46	p_trend_ = 0.33		p_trend_ = 0.96	p_trend_ = 0.98
Daily sunlight exposure by age at exposure							
Teens							
Low	63 (20.9)	27 (13.6)	1.00 (reference)	1.00 (reference)	21 (13.2)	1.00 (reference)	1.00 (reference)
Medium	104 (34.4)	68 (34.2)	1.28 (0.71-2.30)	1.18 (0.62-2.25)	55 (34.6)	1.24 (0.64-2.42)	0.94 (0.45-1.97)
High	135 (44.7)	104 (52.3)	1.38 (0.78-2.43)	1.47 (0.78-2.77)	83 (52.2)	1.43 (0.75-2.73)	1.40 (0.68-2.89)
Twenties							
Low	121 (40.2)	62 (31.0)	1.00 (reference)	1.00 (reference)	53 (33.3)	1.00 (reference)	1.00 (reference)
Medium	114 (37.9)	79 (39.5)	1.20 (0.77-1.88)	1.35 (0.82-2.21)	51 (32.1)	0.82 (0.49-1.37)	0.97 (0.54-1.73)
High	66 (21.9)	59 (29.5)	1.22 (0.73-2.31)	1.31 (0.75-2.31)	55 (34.6)	1.40 (0.80-2.44)	1.56 (0.83-2.91)
Thirties							
Low	152 (51.9)	87 (43.9)	1.00 (reference)	1.00 (reference)	64 (40.3)	1.00 (reference)	1.00 (reference)
Medium	103 (35.2)	63 (31.8)	0.90 (0.58-1.39)	0.98 (0.60-1.58)	62 (39.0)	1.08 (0.66-1.75)	1.36 (0.78-2.37)
High	38 (13.0)	48 (24.2)	1.20 (0.68-2.10)	1.28 (0.69-2.36)	33 (20.8)	1.15 (0.61-2.18)	1.30 (0.63-2.68)
Past 10 years							
Low	126 (57.3)	76 (44.2)	1.00 (reference)	1.00 (reference)	80 (54.1)	1.00 (reference)	1.00 (reference)
Medium	65 (29.5)	59 (34.3)	1.05 (0.64-1.74)	1.15 (0.66-2.01)	40 (27.0)	0.77 (0.45-1.31)	1.13 (0.61-2.10)
High	29 (13.2)	37 (21.5)	1.41 (0.74-2.68)	1.62 (0.79-3.30)	28 (18.9)	1.16 (0.59-2.25)	1.57 (0.73-3.36)

^1^Odds ratios (OR) and 95% confidence intervals (CI) adjusted for age, gender, education, and history of ever smoking.

^2^OR and 95% CI adjusted for age, gender, education, history of ever smoking, cutaneous sensitivity and tanning ability to sunlight exposure.

## Discussion

A clinic based case–control study was conducted to identify associations between patterns and timing of sunlight exposure and two types of NMSC, BCC and SCC. It has been suggested that BCC and SCC risk may differ by the patterns and timing in which sunlight exposure was received. Utilizing similar definitions of sunlight exposure (i.e. number of hours of sunlight exposure to define intermittent and continuous exposure) we investigated multiple measures of sunlight exposure in BCC and SCC simultaneously and did not observe differences in measures of intermittent and continuous patterns of sunlight exposure between the two types of skin cancer. For example, having a job in sun for >10 years and cumulative sunlight exposure in early life were associated with both BCC and SCC. In addition, the appearance of ≥10 moles on the entire body was significantly associated with SCC but not BCC, although the LRT did not demonstrate statistically significant differences in the OR effect sizes in SCC versus BCC.

Findings from previous studies that aimed to quantify the association between the amount of sunlight exposure and NMSC suggested that intermittent sunlight exposure is associated with BCC [24,32] while chronic sunlight exposure is associated with SCC [17,20,33-36]. Two of three previous case–control studies [20,34,37] observed associations between SCC and history of blistering sunburn while no associations have been previously reported with BCC [24,34,37,38]. Blistering sunburn is believed to result from high doses of intense UVR exposure in short increments of time and is therefore considered a measure of intermittency. However, blistering sunburn is also a measure of cutaneous sensitivity to sunlight exposure and may explain the observed associations in our study population for both BCC and SCC when co-factors measuring skin susceptibility to sunlight exposure were excluded from the multivariate models.

It has been estimated that approximately 25% of lifetime sunlight exposure occurs before 18 years of age [39]. Young childhood and adolescence is considered a time period when individuals have greater vulnerability to toxic exposure, such as UVR [39]. Associations with first occurrence of blistering sunburn during childhood or adolescence (age periods prior to skin cancer diagnosis) were similar for BCC and SCC risk in our study population when adjusting for demographic and lifestyle factors but not skin susceptibility factors to sunlight exposure. Among residents of Western Australia, blistering sunburn between 10 to 14 years of age was associated with BCC [24] while sunburn between 35 to 39 years of age was associated with SCC [20]. Many epidemiologic studies have investigated the association between sunlight exposure in early childhood and nevus development and provide evidence that increasing sunlight exposure in early years of life is associated with melanocytic nevus development [26-31]. Since most nevi develop in childhood and early adolescence [26-31] and the number of moles on the body decrease with an increase in age [40-43] their presence in adulthood may be considered an indicator of high UV exposure in childhood. Self-reported presence of ≥10 moles on the entire body were significantly and positively associated with SCC in our study population. Similar results were not observed for BCC. A limited number of studies have reported findings for the association between the presence of moles and NMSC, of which, one case–control study from Western Australia [44] and one prospective cohort study of U.S. male health professionals [45] observed a positive dose–response relationship between an increasing number of moles and BCC. In contrast, among adults from the U.S. [37], the presence of moles was not associated with either BCC or SCC risk.

The current study has some limitations. Clinic based study populations are not necessarily representative samples of the general population. Information on the sub-type of BCC diagnosis (superficial and nodular) was not available for the BCC cases included in the current study population. As reported by Pelucchi et al. [46], the risk of superficial versus nodular BCC may differ by patterns of sunlight exposure. If proven true, this may minimize the differences in the effect sizes observed between BCC and SCC cases in relation to measures of patterns of sunlight exposure. The ability to detect statistically significant associations was limited by the small sample size including participants with a wide age distribution, as well as by exposure variables with multiple strata and adjustment for multiple co-factors. Additionally, sunlight exposure was not assessed at the site of BCC or SCC diagnosis, as done in previous studies [20,24]. Depending on the site of skin cancer diagnosis, this may result in participants underestimating the amount of sunlight exposure to the site of skin cancer diagnosis which, in turn would attenuate the observed differences. The anatomical distributions of BCC and SCC on the face, ears/neck, arms/hands, or other body parts are 76% and 39%, 12% and 14%, 3% and 21%, and 9% and 27%, respectively. Among BCC and SCC cases combined, 60% of skin cancers in our study population occurred on the face. Since the face is chronically exposed to sunlight exposure regardless of the outdoor activity or type of clothing being worn, this could result in cases under-reporting their sunlight exposure. We also did not collect information on sunlight exposure during holidays or recreational activities. It is difficult to compare results across studies for the relationship between sunlight exposure and skin cancer due mainly to inconsistencies and variations in the methods used to measure sunlight exposure.

The questionnaire utilized in the current study includes questions previously validated for use in a skin cancer study conducted in Arizona, US [37]. However, despite the validity, there are additional challenges in exposure ascertainment by use of self-administered questionnaire that should be noted, such as recall bias, misclassification of exposure, and bias due to missing data. Case–control studies are often subject to recall bias because cases tend to think about their exposures more carefully as they might relate to their current cancer diagnosis. In addition, self-reported measures of previous sunlight exposures may result in measurement error from difficulty in remembering habits in the past. However, there is no reason to believe that the type of NMSC (i.e. BCC vs. SCC) would influence patients to think differently about their past sunlight exposure that would affect the OR comparison between BCC and SCC cases. Additionally, no significant differences in age and gender were observed between BCC and SCC cases that completed the study questionnaire and those that did not and further investigations demonstrated no bias due to missing data in the effect measures between patterns and timing of sunlight exposure in NMSC. Unlike previous studies [20,24,36], we measured intermittency of sunlight exposure in the current study by assuming that weekend hours were “non-working” hours for our study population and we were unable to estimate “lifetime” sunlight exposure or consider the amount of ambient solar irradiance received by study participants. Additionally, with regards to occupational sunlight exposure, caution should be taken when interpreting the results. More specifically, having a job in the sun for ≥3 months could involve indoor work up to 9 months of the year. However, as outlined in the introduction, intermittent sunlight exposure has been defined in previous studies as well as in the current study as sunlight exposure received mostly during non-working days (assuming non-working days is ≤2 days per week) or during a traditional 2 day weekend or while vacationing to regions with a higher UV index than an individual’s place of residence. Given this definition, a job in the sun for ≥3 months is indicative of continuous sunlight exposure. However, it is also possible that having a job in the sun for ≥3 months could involve initial intermittent sunlight exposure provided the individual’s occupation included indoor work during the previous 9 months. However, for individuals reporting a job in the sun for ≥3 months for >10 years it is possible to conclude that these individuals have had high levels of continuous sunlight exposure for a minimum of 10 years. Finally, caution should be taken when interpreting the observations between age at first tanning bed use and SCC. Younger age at first tanning bed use was associated with SCC, but not BCC, and older age at first use was not associated with either skin cancer type in our study. While this observation may suggest that sunlight exposure at an earlier age is more important for SCC than BCC risk, it may also be an indicator of higher cumulative lifetime UVR exposure and as previously discussed, it has been hypothesized that continuous, lifelong sunlight exposure increases the risk for SCC.

Strengths of the current study should also be noted; it is the first case–control study to formally evaluate measures of patterns and timing of sunlight exposure in NMSC in a high risk U.S. population as well as to present findings simultaneously for both BCC and SCC, allowing for direct comparisons of patterns and timing of sunlight by skin cancer type. The controls were screened for current signs of BCC and SCC by a nurse practitioner to avoid misclassification of case–control status that may result from self-reported data. This is an important strength of our study as a portion of the screened patients were included as cases.

Understanding how sunlight exposure responses may potentially differ by NMSC type is important for better educating the public in sun safe behaviors. Simply advising a reduction in sunlight exposure will not help reduce the incidence of NMSC if changes in sunlight exposure patterns are related to skin cancer development. For example, applying sunscreen while on vacation may decrease BCC risk associated with intermittent sunlight exposure, but may not impact the risk of SCC, which may be more strongly related with continuous sunlight exposure. Additional studies are needed to highlight similarities and differences in the exposure-response relationship of patterns and timing of sunlight exposure with BCC and SCC. Furthermore, standardized methods for measuring sunlight exposure should be established to enable comparisons across different study populations.

## Abbreviations

NMSC: Non-melanoma skin cancer; BCC: Basal cell carcinoma; SCC: Squamous cell carcinoma; UVR: Ultraviolet radiation; OR: Odds ratio; CI: Confidence interval; U.S.: United States; USF: University of South Florida; D: Dermatology; FM: Family Medicine; LCS: Lifetime Cancer Screening; SPF: Sun protection factor.

## Competing interests

The authors declare that they have no competing interests.

## Authors’ contributions

All authors of this research paper have directly participated in the planning, execution, or analysis of the study and have read an approved the final version submitted. Specifically, DER was the principle investigator that conceived the study, received grant funding and oversaw all study procedures, as well as contributed intellectually to the manuscript. MRI developed the concept of the manuscript, contributed significantly to the organization and writing of the manuscript, and contributed significantly to data collection and data analysis. WW, HGS, and KO contributed intellectually to the manuscript. ARG and VKS contributed to gaining grant funding and significantly contributed intellectually to the manuscript. JLM was the study pathologist and significantly contributed to the end point analysis. RGR, BSC, and NAF significantly contributed to data collection and intellectual review of the manuscript. All authors read and approved the final manuscript.

## Pre-publication history

The pre-publication history for this paper can be accessed here:

http://www.biomedcentral.com/1471-2407/12/417/prepub

## References

[B1] Jemal A Siegel R Ward E Hao Y Xu J Murray T Thun MJ Cancer statistics, 2008 CA Cancer J Clin 2008 58 2 71 96 10.3322/CA.2007.0010 18287387

[B2] Miller DL Weinstock MA Nonmelanoma skin cancer in the United States: incidence J Am Acad Dermatol 1994 30 5 Pt 1 774 778 8176018 10.1016/s0190-9622(08)81509-5

[B3] Weinstock MA Epidemiologic investigation of nonmelanoma skin cancer mortality: the Rhode Island Follow-Back Study J Invest Dermatol 1994 102 6 6S 9S 10.1111/1523-1747.ep12385735 8006441

[B4] Housman TS Feldman SR Williford PM Fleischer AB Jr Goldman ND Acostamadiedo JM Chen GJ Skin cancer is among the most costly of all cancers to treat for the Medicare population J Am Acad Dermatol 2003 48 3 425 429 10.1067/mjd.2003.186 12637924

[B5] Friedman GD Tekawa IS Association of basal cell skin cancers with other cancers (United States) Cancer Causes Control 2000 11 10 891 897 10.1023/A:1026591016153 11142523

[B6] Frisch M Hjalgrim H Olsen JH Melbye M Risk for subsequent cancer after diagnosis of basal-cell carcinoma. A population-based, epidemiologic study Ann Intern Med 1996 125 10 815 821 8928988 10.7326/0003-4819-125-10-199611150-00005

[B7] Frisch M Melbye M New primary cancers after squamous cell skin cancer Am J Epidemiol 1995 141 10 916 922 7741121 10.1093/oxfordjournals.aje.a117358

[B8] Hemminki K Jiang Y Steineck G Skin cancer and non-Hodgkin's lymphoma as second malignancies. markers of impaired immune function? Eur J Cancer 2003 39 2 223 229 10.1016/S0959-8049(02)00595-6 12509955

[B9] Levi F La Vecchia C Te VC Randimbison L Erler G Incidence of invasive cancers following basal cell skin cancer Am J Epidemiol 1998 147 8 722 726 10.1093/oxfordjournals.aje.a009516 9554413

[B10] Levi F Randimbison L La Vecchia C Erler G Te VC Incidence of invasive cancers following squamous cell skin cancer Am J Epidemiol 1997 146 9 734 739 10.1093/oxfordjournals.aje.a009349 9366621

[B11] Milan T Pukkala E Verkasalo PK Kaprio J Jansen CT Koskenvuo M Teppo L Subsequent primary cancers after basal-cell carcinoma: A nationwide study in Finland from 1953 to 1995 Int J Cancer 2000 87 2 283 288 10.1002/1097-0215(20000715)87:2<283::AID-IJC21>3.0.CO;2-I 10861488

[B12] Rosenberg CA Greenland P Khandekar J Loar A Ascensao J Lopez AM Association of nonmelanoma skin cancer with second malignancy Cancer 2004 100 1 130 138 10.1002/cncr.11874 14692033

[B13] Troyanova P Danon S Ivanova T Nonmelanoma skin cancers and risk of subsequent malignancies: a cancer registry-based study in Bulgaria Neoplasma 2002 49 2 81 85 12088110

[B14] Gafa L Filippazzo MG Tumino R Dardanoni G Lanzarone F Dardanoni L Risk factors of nonmelanoma skin cancer in Ragusa, Sicily: A case–control study Cancer Causes Control 1991 2 6 395 399 10.1007/BF00054300 1764564

[B15] Gellin GA Kopf AW Garfinkel L Basal Cell Epithelioma. a Controlled Study of Associated Factors Arch Dermatol 1965 91 38 45 10.1001/archderm.1965.01600070044004 14229596

[B16] Hunter DJ Colditz GA Stampfer MJ Rosner B Willett WC Speizer FE Risk factors for basal cell carcinoma in a prospective cohort of women Ann Epidemiol 1990 1 1 13 23 10.1016/1047-2797(90)90015-K 1669486

[B17] Vitasa BC Taylor HR Strickland PT Rosenthal FS West S Abbey H Ng SK Munoz B Emmett EA Association of nonmelanoma skin cancer and actinic keratosis with cumulative solar ultraviolet exposure in Maryland watermen Cancer 1990 65 12 2811 2817 10.1002/1097-0142(19900615)65:12<2811::AID-CNCR2820651234>3.0.CO;2-U 2340474

[B18] Ferreira FR Nascimento LF Rotta O Risk factors for nonmelanoma skin cancer in Taubate, Sao Paulo, Brazil: a case–control study Rev Assoc Med Bras 2011 57 4 424 430 21876926

[B19] Simic D Situm M Marijanovic I Hadzigrahic N Most common skin tumours in correlation with solar ultraviolet radiation in the area of West Herzegovina Coll Antropol 2011 35 4 1129 1134 22397249

[B20] English DR Armstrong BK Kricker A Winter MG Heenan PJ Randell PL Case–control study of sun exposure and squamous cell carcinoma of the skin Int J Cancer 1998 77 3 347 353 10.1002/(SICI)1097-0215(19980729)77:3<347::AID-IJC7>3.0.CO;2-O 9663594

[B21] Gallagher RP Hill GB Bajdik CD Coldman AJ Fincham S McLean DI Threlfall WJ Sunlight exposure, pigmentation factors, and risk of nonmelanocytic skin cancer. II. Squamous cell carcinoma Arch Dermatol 1995 131 2 164 169 10.1001/archderm.1995.01690140048007 7857112

[B22] Gallagher RP Hill GB Bajdik CD Fincham S Coldman AJ McLean DI Threlfall WJ Sunlight exposure, pigmentary factors, and risk of nonmelanocytic skin cancer. I. Basal cell carcinoma Arch Dermatol 1995 131 2 157 163 10.1001/archderm.1995.01690140041006 7857111

[B23] Gallagher RP Spinelli JJ Lee TK Tanning beds, sunlamps, and risk of cutaneous malignant melanoma Cancer Epidemiol Biomarkers Prev 2005 14 3 562 566 10.1158/1055-9965.EPI-04-0564 15767329

[B24] Kricker A Armstrong BK English DR Heenan PJ Does intermittent sun exposure cause basal cell carcinoma? a case–control study in Western Australia Int J Cancer 1995 60 4 489 494 10.1002/ijc.2910600411 7829262

[B25] Rollison DE Iannacone MR Messina JL Glass LF Giuliano AR Roetzheim RG Cherpelis BS Fenske NA Jonathan KA Sondak VK Case–control study of smoking and non-melanoma skin cancer Cancer Causes Control 2012 23 2 245 254 10.1007/s10552-011-9872-y 22101452

[B26] Bauer J Buttner P Wiecker TS Luther H Garbe C Risk factors of incident melanocytic nevi: a longitudinal study in a cohort of 1,232 young German children Int J Cancer 2005 115 1 121 126 10.1002/ijc.20812 15645451

[B27] Darlington S Siskind V Green L Green A Longitudinal study of melanocytic nevi in adolescents J Am Acad Dermatol 2002 46 5 715 722 10.1067/mjd.2002.120931 12004313

[B28] Dulon M Weichenthal M Blettner M Breitbart M Hetzer M Greinert R Baumgardt-Elms C Breitbart EW Sun exposure and number of nevi in 5- to 6-year-old European children J Clin Epidemiol 2002 55 11 1075 1081 10.1016/S0895-4356(02)00484-5 12507670

[B29] Harrison SL MacLennan R Buettner PG Sun exposure and the incidence of melanocytic nevi in young Australian children Cancer Epidemiol Biomarkers Prev 2008 17 9 2318 2324 10.1158/1055-9965.EPI-07-2801 18768500

[B30] Oliveria SA Satagopan JM Geller AC Dusza SW Weinstock MA Berwick M Bishop M Heneghan MK Halpern AC Study of Nevi in Children (SONIC): baseline findings and predictors of nevus count Am J Epidemiol 2009 169 1 41 53 19001133 10.1093/aje/kwn289 PMC2720704

[B31] Pettijohn KJ Asdigian NL Aalborg J Morelli JG Mokrohisky ST Dellavalle RP Crane LA Vacations to waterside locations result in nevus development in Colorado children Cancer Epidemiol Biomarkers Prev 2009 18 2 454 463 10.1158/1055-9965.EPI-08-0634 19190148 PMC2999580

[B32] Kricker A Armstrong BK English DR Heenan PJ A dose–response curve for sun exposure and basal cell carcinoma Int J Cancer 1995 60 4 482 488 10.1002/ijc.2910600410 7829261

[B33] Aubry F MacGibbon B Risk factors of squamous cell carcinoma of the skin. A case–control study in the Montreal region Cancer 1985 55 4 907 911 10.1002/1097-0142(19850215)55:4<907::AID-CNCR2820550433>3.0.CO;2-5 3967184

[B34] Han J Colditz GA Hunter DJ Risk factors for skin cancers: a nested case–control study within the Nurses' Health Study Int J Epidemiol 2006 35 6 1514 1521 10.1093/ije/dyl197 16943234

[B35] Perea-Milla Lopez E Minarro-Del Moral RM Martinez-Garcia C Zanetti R Rosso S Serrano S Aneiros JF Jimenez-Puente A Redondo M Lifestyles, environmental and phenotypic factors associated with lip cancer: a case–control study in southern Spain Br J Cancer 2003 88 11 1702 1707 10.1038/sj.bjc.6600975 12771984 PMC2377138

[B36] Rosso S Zanetti R Martinez C Tormo MJ Schraub S Sancho-Garnier H Franceschi S Gafa L Perea E Navarro C The multicentre south European study 'Helios'. II: Different sun exposure patterns in the aetiology of basal cell and squamous cell carcinomas of the skin Br J Cancer 1996 73 11 1447 1454 10.1038/bjc.1996.275 8645596 PMC2074492

[B37] Foote JA Harris RB Giuliano AR Roe DJ Moon TE Cartmel B Alberts DS Predictors for cutaneous basal- and squamous-cell carcinoma among actinically damaged adults Int J Cancer 2001 95 1 7 11 10.1002/1097-0215(20010120)95:1<7::AID-IJC1001>3.0.CO;2-X 11241303 PMC2637530

[B38] Boyd AS Shyr Y King LE Jr Basal cell carcinoma in young women: an evaluation of the association of tanning bed use and smoking J Am Acad Dermatol 2002 46 5 706 709 10.1067/mjd.2002.120467 12004311

[B39] Balk SJ Ultraviolet Radiation: A Hazard to Children and Adolescents Pediatrics 2011 127 3 e791 e817 10.1542/peds.2010-3502 21357345

[B40] Lasithiotakis KG Kokolakis A Giannikaki E Krasagakis K Tosca A Factors associated with the prevalence of atypical nevus in a Mediterranean pigmented skin lesion clinic Melanoma Res 2011 21 5 469 473 10.1097/CMR.0b013e32834941f9 21760555

[B41] MacKie RM English J Aitchison TC Fitzsimons CP Wilson P The number and distribution of benign pigmented moles (melanocytic naevi) in a healthy British population Br J Dermatol 1985 113 2 167 174 10.1111/j.1365-2133.1985.tb02060.x 4027184

[B42] Silva Idos S Higgins CD Abramsky T Swanwick MA Frazer J Whitaker LM Blanshard ME Bradshaw J Apps JM Bishop DT Overseas sun exposure, nevus counts, and premature skin aging in young English women: a population-based survey J Invest Dermatol 2009 129 1 50 59 10.1038/jid.2008.190 18615111

[B43] Zalaudek I Schmid K Marghoob AA Scope A Manzo M Moscarella E Malvehy J Puig S Pellacani G Thomas L Frequency of dermoscopic nevus subtypes by age and body site: a cross-sectional study Arch Dermatol 2011 147 6 663 670 10.1001/archdermatol.2011.149 21690528

[B44] Kricker A Armstrong BK English DR Heenan PJ Pigmentary and cutaneous risk factors for non-melanocytic skin cancer–a case–control study Int J Cancer 1991 48 5 650 662 10.1002/ijc.2910480504 2071226

[B45] van Dam RM Huang Z Rimm EB Weinstock MA Spiegelman D Colditz GA Willett WC Giovannucci E Risk factors for basal cell carcinoma of the skin in men: results from the health professionals follow-up study Am J Epidemiol 1999 150 5 459 468 10.1093/oxfordjournals.aje.a010034 10472945

[B46] Pelucchi C Di Landro A Naldi L La Vecchia C Risk factors for histological types and anatomic sites of cutaneous basal-cell carcinoma: an italian case–control study J Invest Dermatol 2007 127 4 935 944 10.1038/sj.jid.5700598 17068478

